# TWINKLE and Other Human Mitochondrial DNA Helicases: Structure, Function and Disease

**DOI:** 10.3390/genes11040408

**Published:** 2020-04-09

**Authors:** Bradley Peter, Maria Falkenberg

**Affiliations:** Department of Medical Biochemistry and Cell Biology, University of Gothenburg, P.O. Box 440, SE405 30 Gothenburg, Sweden

**Keywords:** mitochondria, mtDNA, replication, PEO, TWINKLE

## Abstract

Mammalian mitochondria contain a circular genome (mtDNA) which encodes subunits of the oxidative phosphorylation machinery. The replication and maintenance of mtDNA is carried out by a set of nuclear-encoded factors—of which, helicases form an important group. The TWINKLE helicase is the main helicase in mitochondria and is the only helicase required for mtDNA replication. Mutations in TWINKLE cause a number of human disorders associated with mitochondrial dysfunction, neurodegeneration and premature ageing. In addition, a number of other helicases with a putative role in mitochondria have been identified. In this review, we discuss our current knowledge of TWINKLE structure and function and its role in diseases of mtDNA maintenance. We also briefly discuss other potential mitochondrial helicases and postulate on their role(s) in mitochondria.

## 1. Overview of mtDNA Replication

Mitochondria contain their own circular genome of 16,569 bp (mtDNA), comprising a light (L) strand and a heavy (H) strand, each containing their own origins of replication (OriH/OriL) and promoters for transcription (heavy strand promoter/HSP and light strand promoter/LSP). In humans, mtDNA contains 37 genes encoding 13 proteins of the oxidative phosphorylation system, 2 ribosomal RNAs (12S and 16S rRNA) and 22 transfer RNA molecules needed for translation of mitochondrial proteins. MtDNA is replicated and maintained by a set of nuclear-encoded proteins which are distinct from those involved in nuclear DNA replication. The minimal mitochondrial replisome comprises (1) the replicative DNA polymerase gamma (POLγ), which is a heterotrimer of the catalytic A subunit and two accessory B subunits [1,2]; (2) the replicative hexameric helicase TWINKLE [3]; (3) the mitochondrial single-stranded DNA-binding protein (mtSSB) [3,4,5]; and (4) the mitochondrial RNA polymerase (POLRMT) [6,7]. Together, these form a processive replisome capable of synthesising DNA molecules >16 kb in length [5]. Defects in mtDNA replication/maintenance due to mutations in these proteins are associated with a number of pathologies.

**Figure 1:**
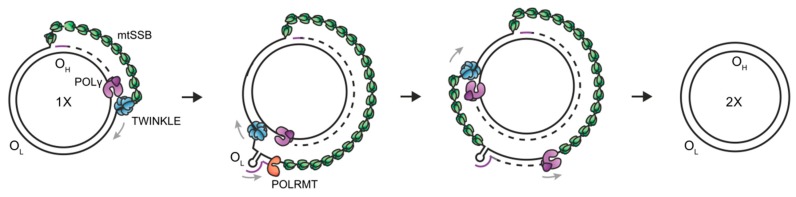
Strand displacement model of mtDNA replication. Replication of mtDNA is initiated at the heavy strand origin of replication (O_H_) by mtSSB, POLγ and TWINKLE and proceeds unidirectionally to produce the full-length nascent H-strand. When the replisome passes the light strand origin of replication (O_L_), a stem-loop structure is formed from which POLRMT can initiate the synthesis of the lagging strand primer. Synthesis of the two strands proceeds in a continuous manner to produce two mtDNA molecules.

Although the mechanism of mtDNA replication is still debated, the generally accepted mechanism is the strand displacement model [8]. Here, both the L-strand and the H-strand DNA synthesis proceeds continuously and without the formation of Okazaki fragments (Figure 1) [9]. Briefly, the synthesis of the nascent H-strand is initiated at OriH, with TWINKLE moving on the parental H-strand ahead of POLγ and mtSSB binding to the displaced parental H-strand [10,11]. When the replisome reaches OriL, the single-stranded parental H-strand folds into a stem-loop structure from which primer synthesis for L-strand replication is initiated by POLRMT. After ~25 nt, POLRMT is replaced by POLγ and nascent H-strand and L-strand synthesis continues until the two replication events have reached full circle. Since L-strand synthesis utilizes a single-stranded template, the TWINKLE helicase is only required for H-strand synthesis where it is involved in duplex unwinding and fork progression.

TWINKLE is the sole replicative helicase in mitochondria. However, a number of additional helicases performing diverse functions have also been suggested to be mitochondrial. Although TWINKLE remains the most well-studied mitochondrial helicase, the need for helicase activity extends beyond solely replicating mtDNA and includes roles in mtDNA damage repair pathways and RNA metabolism. Here, we present a summary of these mitochondrial helicases with a focus on TWINKLE, its role in mitochondria and associated disease-causing mutations.

## 2. TWINKLE Helicase

### 2.1. Structure and Helicase Activity

#### 2.1.1. Structure and Domain Organization

Helicases are a diverse group of motor proteins that utilize the energy from nucleotide triphosphate (NTP) hydrolysis to catalyze the unwinding of duplex DNA/RNA. They are involved in almost all nucleic acid transactions, including DNA replication, transcription, translation, recombination and DNA repair (reviewed in [12,13,14,15]). Based on structure and sequence similarities, helicases can be classified into six superfamilies (SF1–6) [14,16]. TWINKLE is part of the SF4 superfamily and is closely related to the bacteriophage T7 gene protein 4 (gp4) primase-helicase [17].

SF4 helicases are ring shaped and share five conserved helicase motifs: (1) the H1/Walker A motif, which stabilizes the NTP phosphate; (2) H1a, which is involved in NTP binding/hydrolysis; (3) the H2/Walker B motif, which contains an arginine finger and a base stack residue required for positioning and stabilizing the bound NTP as well as stabilizing a bound Mg2+ ion; (4) H3, which with H1a is involved in NTP binding/hydrolysis; and (5) H4, which contributes to DNA binding [14,15,18,19,20,21]. Furthermore, the C-terminal region of many SF4 helicases is required for interactions with other factors in the replisome [22,23,24]. 

**Figure 2:**
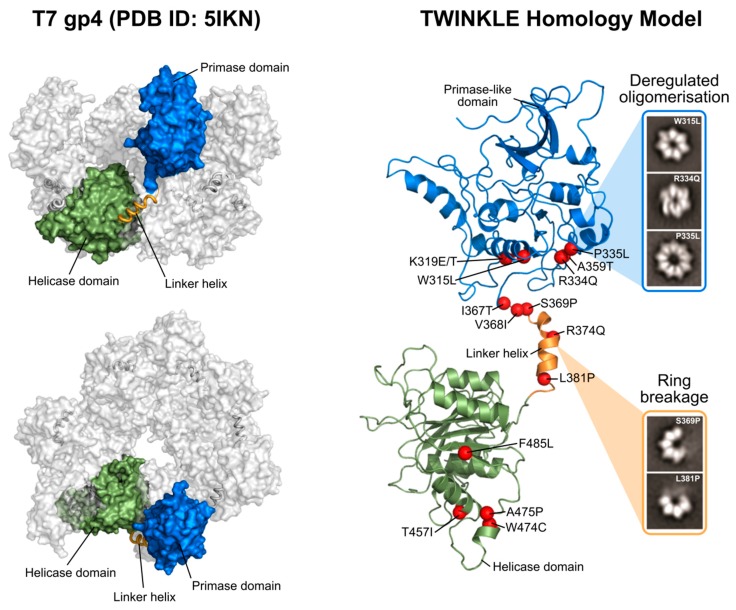
Structure and domain organization of TWINKLE and T7 gp4. (**Left panel**) Top and side views of the heptameric T7 gp4 protein (PDB ID: 5IKN). The helicase (green) and primase (blue) domains as well as the linker helix (orange) are shown. (**Right panel**) Homology model of a TWINKLE monomer comprising a non-functional primase-like domain (blue) connected to a conserved helicase domain (green) by a flexible linker helix (orange). The positions of disease-causing mutations for which in vitro biochemical data is available are shown as red spheres. Single-particle negative stain images are also shown for selected disease variants, highlighting the effects of these mutations on TWINKLE oligomerization.

TWINKLE, in addition to these shared sequence motifs, is hexameric and each 72 kDa monomer is comprised of an N-terminal domain (NTD) and C-terminal domain (CTD) joined by a flexible linker helix (Figure 2). The CTD is highly conserved and contains the five aforementioned SF4 motifs, including the Walker A and B motifs, which provide the catalytic residues for NTP hydrolysis [14]. The incoming NTP binds in a cleft between two neighboring CTDs, effectively linking the subunits and allowing for coordinated movement following NTP hydrolysis [18]. In T7 gp4, the NTD functions as a primase in addition to its role as a helicase [18]. Here, a zinc-binding domain (ZBD) binds to ssDNA and transfers it to the RNA polymerase domain (RPD), which subsequently catalyzes NTP addition [17,25,26]. The NTD of TWINKLE, however, has lost the primase activity as a result of divergent evolution [25,27,28]. While the TWINKLE NTD contains the conserved primase motifs II-VI characteristic of other TOPRIM (topoisomerase primase fold) proteins, it lacks three of the four conserved cysteine residues found in the ZBD (motif I) needed for DNA binding and primase activity [17,25,29,30]. In mammalian mitochondria, this primase function has instead been taken over by the mitochondrial RNA polymerase POLRMT [7]. While the TWINKLE NTD is not strictly required for replisome function per se, it is needed for the synthesis of long DNA products (> 1 kb in size) and truncations in this region are associated with impaired ssDNA binding, helicase activity and processivity [31]. In addition, the C-terminal region of the TWINKLE NTD contributes to oligomer stability in vitro [17,31].

As a member of the SF4 helicase superfamily, TWINKLE functions as a ring-shaped helicase. As such, the TWINKLE monomers must come together to form higher-order oligomers. There are no crystal structures available of TWINKLE but several structures of the homologous T7 gp4 as well as a low-resolution cryo-EM map of TWINKLE have been published [18,32,33,34]. In these structures, both hexameric and heptameric rings are visible (Figure 2) and adopt a compact two-tier arrangement surrounding a central pore. The linker helix between the NTD and CTD plays an important role in oligomerization by forming a stable helix bundle at the surface of the CTD of the neighboring subunit (Figure 2) [35]. This interaction between the linker helix and neighboring CTD not only contributes to oligomer stability, but also couples the subunits in such a way that, upon NTP hydrolysis, the CTDs rotate and shift in relation to one another to provide the mechanical force required for DNA unwinding [15,18,36]. Indeed, the linker region of T7 gp4 is crucial for both oligomerization and helicase activity [37,38] and the importance of this region is augmented by the fact that many disease-causing TWINKLE mutations are positioned within the linker helix and its vicinity (Figure 2, Figure 4 and Section 2.2; [39,40,41]).

The oligomeric state of TWINKLE has also been shown to be modulated by salt concentration as well as the presence of Mg^2+^ and NTPs, enabling conversion between hexameric and heptameric arrangements [27,42]. Such transitions have also been observed in T7 gp4 [18,33] and some SF6 helicases [14]. The significance of such an oligomeric state exchange is addressed in Section 2.1.2. In addition, the predominantly electrostatic interactions identified between the NTD and CTD in the TWINKLE cryo-EM map render the activity of TWINKLE potentially tunable in response to environmental factors [34]. Interpretation of the significance of this low resolution TWINKLE cryo-EM map is, however, made difficult by the fact that (1) it was obtained at high salt (1-1.5 M) and using chemical fixation and (2) the map is not available on public databases such as The Electron Microscopy Data Bank (EMDB). Further structural studies of TWINKLE should therefore be focused on acquiring high-resolution data using conditions which more closely mimic the physiological environment in which TWINKLE exists. This is easier said than done, given that the poor solubility of TWINKLE in low-salt buffers makes structural analysis difficult [27].

#### 2.1.2. TWINKLE as the Sole Replicative Helicase

Numerous studies have shown that TWINKLE, while not the only mitochondrial helicase, is the sole replicative helicase required for mtDNA maintenance. In vivo mouse models have shown that TWINKLE is essential for embryonic development [43]. Knockdown of TWINKLE is embryonically lethal and associated with severe mtDNA depletion, impaired mtDNA expression and respiratory chain deficiency [43]. This seems to confirm that TWINKLE is essential for mtDNA replication and cannot be substituted by other mitochondrial helicases.

**Figure 3:**
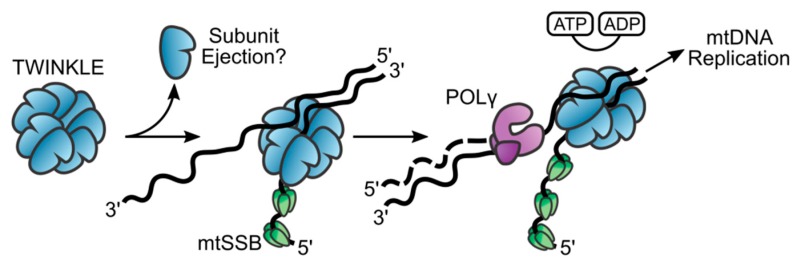
Helicase activity of TWINKLE. TWINKLE is the sole replicative helicase in mitochondria. It is proposed that heptameric TWINKLE ejects a subunit upon binding to ssDNA and performs ATP-dependent 5ʹ–3ʹ unwinding of DNA. This enables POLRMT to synthesize RNA primers on which POLγ can extend, allowing for leading-strand replication. The displaced ssDNA is coated by mtSSB until it is displaced by the lagging-strand replication machinery.

Functionally, TWINKLE localizes to the mitochondrial nucleoid, where it acts as a 5ʹ–3ʹ DNA helicase (Figure 3) [3,44]. In vitro, it interacts with a variety of DNA substrates including both linear and circular ssDNA and linear dsDNA, while showing a greater affinity for dsDNA [3,31,45]. In order to efficiently initiate DNA unwinding, TWINKLE requires a fork structure comprising a single-stranded 5ʹ DNA end and a short 3ʹ tail [3,46]. In vivo, initiation of DNA synthesis at OriH requires TWINKLE to load onto a closed circular mtDNA molecule. Studies by our laboratory have shown that this is possible without the need for a specialized helicase-loading factor and that TWINKLE can support initiation of DNA replication on closed circular dsDNA in vitro [45]. This is not to discount the possible existence of a loading factor in vivo, although no such protein has yet been identified in mitochondria.

The exact mechanism by which TWINKLE loads onto the DNA is still unclear. However, the dynamic nature of TWINKLE’s oligomeric state- shifting from heptamers to hexamers- may provide some insight [27]. A previous study on T7 gp4 postulates that a switch from heptamer to hexamer may provide a ring-opening mechanism for loading onto ssDNA (Figure 3) [47]. Similarly, a recently published study has shown that TWINKLE oligomers exist in both closed- and open-ring conformations and load onto ssDNA in an open-ringed conformation [48]. Crystal structures of T7 gp4 as well as EM models of TWINKLE show that the central channel of the hexamer is wide enough to accommodate ssDNA but not dsDNA [18,34]. However, elucidation of a high-resolution structure comprising TWINKLE bound to and actively replicating a DNA substrate is necessary in order to understand this process.

Once loaded onto the DNA, TWINKLE is committed to replication initiation. Although several models for DNA unwinding exist, the generally accepted model for most hexameric helicases, including T7 gp4, is the steric-exclusion model [14,19]. In this model, the helicase binds to one of the strands of the dsDNA and threads it through its central pore while simultaneously forcing the other strand outside of the hexamer by steric exclusion and specific contacts with the outer surface of the helicase (Figure 3) [19,49]. Two ssDNA-binding sites have been identified in TWINKLE: (1) ssDNA in the central pore, and (2) ssDNA on the outer surface of the helicase ring [50]. This is consistent with the steric exclusion model, which requires ssDNA-binding sites both within the central pore and on the helicase surface. 

#### 2.1.3. Additional Proposed Functions of TWINKLE

In addition to its helicase activity, it has been demonstrated that TWINKLE possesses NTP-dependent strand annealing and strand exchange activity [50,51,52]. The physiological significance of such activity is not clear, but it is suggested to play a role in mtDNA repair and/or in recombination of mtDNA. mtDNA damage repair pathways remain poorly understood and only base excision repair activities have been strongly elucidated thus far [53,54,55]. The oxidative environment of the mitochondrial matrix coupled with the inherent error rate of POLγ results in mtDNA with a significantly higher mutation rate compared to the nuclear genome [56,57,58,59]. Damage to mtDNA is associated with mtDNA deletions and premature aging [60,61,62,63,64]. Chen et al. ([65]) have proposed a role for TWINKLE in recombination-mediated double stranded break (DSB) repair. However, it is still debated whether classical recombination and DSB repair exist in human mitochondria [66].

Furthermore, TWINKLE probably plays a role in the regulation of mtDNA replication/maintenance. Nearly 95% of all replication events initiated at OriH are prematurely terminated after ~650 nucleotides, forming a triple-stranded displacement loop (D-loop) structure [67,68]. It is theorized that this pre-termination acts as a switch between abortive and full-length mtDNA replication and thus can regulate mtDNA levels in the cell. TWINKLE has been suggested to play a key role in this switch. In support of this, mouse genetic experiments demonstrate that TWINKLE is important for mtDNA copy number control since TWINKLE levels correlate nicely with mtDNA copy number [43,69,70]. This suggests that TWINKLE may serve a dual role in mitochondria as both the replicative DNA helicase and a key regulator of mtDNA maintenance. For further reading, an excellent review is available discussing the function of the mitochondrial D-loop structure [71].

#### 2.1.4. TWINKLE in the Context of the Replisome

In vivo, TWINKLE does not exist in isolation but rather forms part of a larger replisome comprising the DNA polymerase gamma holoenzyme (POLγ), mitochondrial single-stranded DNA-binding protein (mtSSB) and the mitochondrial RNA polymerase (POLRMT). Structural evidence for a direct physical interaction between TWINKLE and the rest of the replisome is lacking, although it is known that they colocalize in mitochondrial nucleoids (reviewed by [72,73]). In vitro biochemistry by our lab and others has demonstrated a functional link between these components. Although no high-resolution structure of the mitochondrial replisome is available, both crystal structures and cryo-EM maps of the T7 replisome have recently been solved [74,75,76]. Given the high degree of similarity between the mitochondrial and T7 replisomes, these structures provide a starting point for probing interactions between TWINKLE and POLγ, mtSSB and POLRMT.

Leading-strand DNA synthesis requires both physical and functional coupling between the helicase and the leading-strand polymerase [5,77]. Although the exact mechanism of this coupling is unclear, it is commonly believed that the helicase is the driving motor behind which the polymerase either pushes or prevents backward slippage of the helicase [77]. In T7, the gp4 helicase has been shown to interact with a basic loop in the DNA polymerase via an acidic C-terminal tail in the helicase domain [23,24,74,78]. This region is partly conserved in TWINKLE. Indeed, a TWINKLE mutant lacking the NTD but preserving this C-terminal tail was still capable of stimulating POLγ activity [31]. Like other replication complexes, this interaction between TWINKLE and POLγ increases both the speed of DNA unwinding and processivity [5,79,80]. The cryo-EM study by Gao et al. ([76]) reported several different structures showing different orientations of the gp4 helicase with respect to the polymerase, suggesting that these helicase-polymerase interactions are highly dynamic. Interestingly, the T7 gp4 helicase can load and exchange multiple polymerase molecules via its acidic C-terminal tail [81]. This is thought to aid processivity as well as bypassing damaged DNA, although whether or not TWINKLE utilizes a similar approach is unknown [24]. Despite the sequence and structure similarities between TWINKLE/POLγ and their T7 counterparts, differences in the way in which DNA is replicated in mitochondria and T7 bacteriophages (eg: strand displacement model vs coupled leading and lagging strand replication) may limit the extent to which TWINKLE-POLγ interactions can be modelled on the T7 replisome.

Since initiation of mtDNA replication requires RNA primers, helicase–primase interactions likely play a role in regulating mtDNA levels in cells. In T7, the primase and helicase activities are both carried out by gp4 and as such the primase is always physically associated with the replicative helicase. In TWINKLE, however, this function has been lost and instead POLRMT produces the RNA primers needed for replication initiation. As such, POLRMT–TWINKLE interactions cannot be modelled on the T7 replisome. In the T4 and *Escherichia coli* replisomes, the primase is closely associated with the replicative helicase where it enhances helicase processivity by stabilizing DNA binding and helicase hexamerization [82,83]. Although TWINKLE is capable of forming hexamers in the absence of POLRMT, such an interaction may play a similar role in enhancing TWINKLE processivity in vivo.

In addition, extensive biochemical evidence exists to suggest a direct physical interaction between TWINKLE and mtSSB [3,84]. MtSSB stimulates TWINKLE helicase activity, but this effect is also seen when using closely related *Drosophila melanogaster* mtSSB and *E. coli* SSB proteins [84]. Alanine substitution and deletion studies highlight loops 1,2 and 4,5-2 in mtSSB as potentially important for interaction with TWINKLE [84]. In addition, atomic force microscopy (AFM) studies have recently shown that mtSSB can influence the way in which TWINKLE binds to DNA [48]. Physical interactions between replicative helicases and ssDNA-binding proteins have been observed in other systems [85,86,87]. In addition, DNA replication assays combining mutant TWINKLE variants with mtSSB and POLγ suggest that disease-causing mutations have a strong negative effect on these protein-protein interactions (see Section 2.2) [28,39]. Newly reported disease-causing mutations in mtSSB could also help shed light on these interactions and provide evidence for specific functional and physical interactions between TWINKLE and mtSSB [88,89].

Lastly, it has been reported that TWINKLE and other components of the replisome localize to and are active on the inner mitochondrial membrane (IMM) [90]. Although mtDNA has been shown to associate with the IMM [91], TWINKLE does not contain any predicted transmembrane helices. In addition, the methods used by Rajala et al. ([90]) were based on TWINKLE solubility. It is well-known that TWINKLE solubility is highly dependent on environmental composition, particularly if present at high concentrations [27,42]. This, coupled with our own and others’ data showing that TWINKLE is active in the absence of a membrane environment in vitro, suggests that membrane binding is not strictly necessary but may occur in vivo. Co-localization of the replication machinery in mitochondrial nucleoids has, however, been proposed previously [72,73], as has regulation of mtDNA distribution by the outer mitochondrial membrane (OMM) [92,93]. It follows, then, that TWINKLE activity may be influenced to some extent by the IMM. Future experiments will hopefully attempt to clarify this role of the mitochondrial membrane in mtDNA maintenance.

### 2.2. Disease

**Figure 4:**
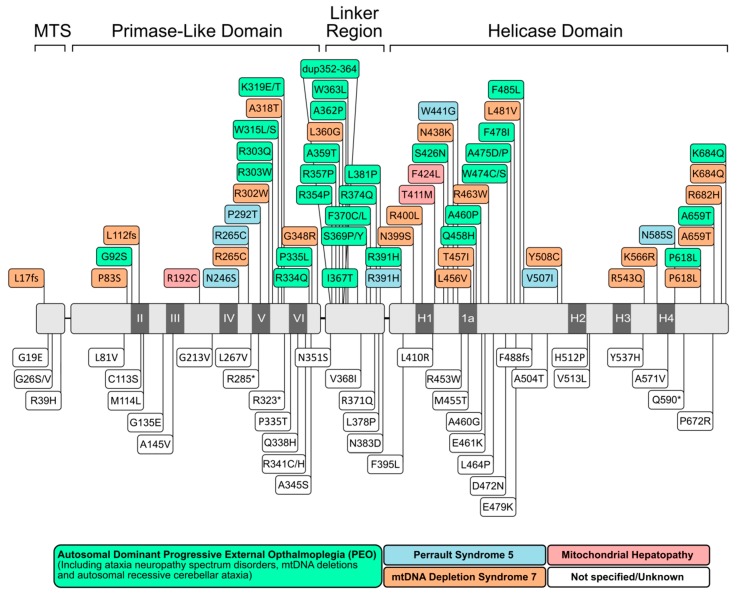
Disease-causing mutations and polymorphisms in the TWINKLE helicase. The domain organization of TWINKLE containing the conserved primase (II–VI) and helicase (H1–H4) motifs is shown. MTS—mitochondrial targeting sequence. The list of mutations was generated based on reported mutations and polymorphisms in the online Mendelian inheritance in man/OMIM database [102]. The upper region shows all reported mutations associated with disease while the lower region shows reported mutations for which a disease phenotype is uncertain or has not yet been described. Note the clustering of disease-causing mutations in the primase-like domain and linker helix regions. Green—adPEO and other ataxia neuropathy spectrum disorders (MIRAS, SANDO). Blue—Perrault syndrome. Pink—mitochondrial hepatopathy. Orange—mtDNA depletion syndrome (including IOSCA). White—not specified/not yet linked to disease.

Mammalian mtDNA encodes key components of the oxidative phosphorylation (OXPHOS) system. Unsurprisingly, defects in mtDNA maintenance are associated with impaired respiratory function and lead to numerous mitochondrial and age-associated diseases in humans [58,94]. MtDNA diseases can be broadly subdivided into two groups: (1) those caused by primary mutations in the mtDNA itself, and (2) those caused by mutations in the nuclear-encoded mtDNA replication/maintenance machinery [40,95]. Here, we will focus on mtDNA maintenance disorders, with several reviews available elsewhere on primary mtDNA mutations in mitochondrial disease [58,96]. Defects in mtDNA maintenance proteins typically result in secondary multiple deletions, duplications or depletion of mtDNA, and subsequent mitochondrial dysfunction and manifest with a broad spectrum of symptoms. Missense mutations in TWINKLE are associated with a number of mitochondrial disorders, including adult-onset progressive external ophthalmoplegia (PEO), mtDNA depletion syndromes (MDSs), Perrault syndrome, infantile-onset spinocerebellar ataxia (IOSCA) and other ataxia neuropathies (Figure 4) [17,97,98,99,100,101].

#### 2.2.1. Progressive External Ophthalmoplegia (PEO)

TWINKLE was originally discovered as the causative gene of autosomal dominant PEO [17]. To date, upwards of 40 point mutations associated with the disease have been discovered in the TWNK gene encoding TWINKLE (Figure 4). This neurodegenerative disease primarily affects the muscles controlling eye movement [103]. These mutant variants show impaired helicase activity, which is thought to stall mtDNA replication and result in the slow accumulation of large mtDNA deletions over time [104,105,106]. Our laboratory and others have extensively studied the effects of many of these TWINKLE mutations both in vitro and in vivo [28,35,39]. Mutations associated with PEO are distributed throughout the entire protein but are particularly prevalent in the NTD and linker helix (Figure 2 and Figure 4).

**Figure 5:**
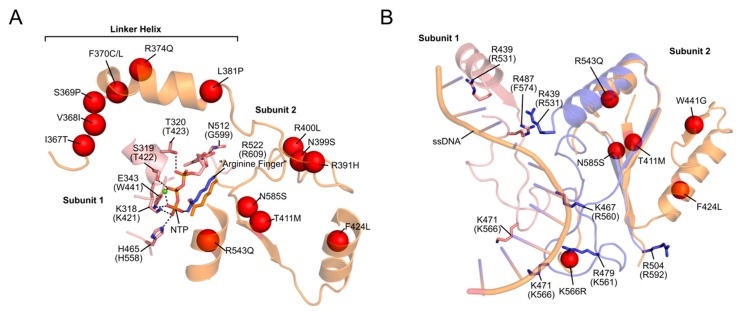
Disease-causing mutations in the NTP-binding and ssDNA-binding regions of TWINKLE. Disease-causing mutations in TWINKLE are often associated with defective ATPase and ssDNA-binding activities, leading to impaired helicase activity and subsequent stalling at the replication fork. The TWINKLE CTD (orange) was aligned to the T7 gp4 CTD (grey/blue; PDB ID: 6N9V). In cases where the T7 residues are shown, the equivalent residue number in TWINKLE is given in parentheses. (**A**) The residues involved in NTP binding/hydrolysis are shown, as are nearby (<10 Å) disease-causing mutations (red spheres). (**B**) Residues involved in ssDNA binding. Both T7 gp4 and TWINKLE possess a conserved set of positively-charged amino acids that form a ssDNA interaction interface. Nearby (<10 Å) disease-causing mutations are indicated (red spheres).

Based on the T7 gp4, the linker helix likely makes intermolecular contacts both with the primase-like domain and with the helicase domain of the neighbouring monomer [35,39,76]. As such, it likely plays a role both in hexamer formation and helicase activity, much like its T7 counterpart [37,107]. Consistent with this role, we showed that PEO mutations in the linker region (A359T, I367T, S369P, R374Q and L381P) disrupt protein hexamerization, reduce ATP hydrolysis and abolish DNA helicase activity [35,39]. The SF4 family of helicases require correct oligomerization and flexibility within the linker region for NTP hydrolysis and helicase activity [108,109]. The proximity of the linker helix to the NTP-binding site is shown in Figure 5. ATP binds at the interface between two monomers, created by amino acids from the linker region and the N-terminal region of the helicase domain. The resulting loss of rotational freedom of the individual helicase subunits due to linker helix mutations would therefore likely result in the uncoupling of NTP hydrolysis to the conformational changes required for DNA propulsion. Mutations in the NTD (W315L, K319T, R334Q, and P335L) are also associated with decreased ATPase activity, impaired ssDNA binding and cannot support wild-type levels of DNA replication in vitro [28]. An interesting observation by Peter et al. ([35]) was a W315L variant which existed almost exclusively as a heptamer, rather than the usual mix of hexamers and heptamers. This was accompanied by poor binding to ssDNA. According to the subunit ejection model (see Section 2.1.2), heptameric helicases can eject a subunit, thereby opening the helicase ring and loading an active hexamer onto ssDNA [47]. If this model is valid for TWINKLE, then the shift in oligomer population due to this mutation would leave the helicase unable to load onto mtDNA. In contrast to the NTD and linker helix, only a handful of mutations in the CTD associated with PEO have been reported (Figure 4) [110]. Given that the CTD is predominantly responsible for helicase activity, it is not surprising that deleterious mutations in this domain are seldom propagated. It is this domain which, with the linker helix, binds to and hydrolyses NTPs (Figure 5). In addition, an alignment of a TWINKLE monomer with the T7 replisome shows that the CTD may also be involved in ssDNA binding (Figure 5). In particular, K566 (K471 in T7 gp4) interacts with the ssDNA and a K566R mutation has been linked to PEO (Figure 5). Two additional reported mutations (R682H and K684Q) localize to the C-terminal tail which, in T7 gp4, is involved in interactions with the DNA polymerase (see Section 2.1.4) [22,23]. Although no biochemical data is available for these mutants, it is reasonable to suppose that replisome integrity might be compromised as a result of impaired helicase–polymerase interactions. Equivalent C-terminal mutations in *D. melanogaster* have been analyzed in vitro, although these studies showed disparate results regarding the severity of the mutations [30,110,111]. It should be noted that the K566R and R682H mutations are carried at a heterozygous state by several control individuals according to the gnomAD database [112], complicating their association with adPEO (although they may play a role in a recessive form of the disease as well as in other mtDNA depletion syndromes).

Available data from in vivo studies confirms our observations from in vitro analyses, with overexpression of PEO TWINKLE variants causing stalling of mtDNA replication and accumulation of dsDNA bubble and y-shaped replication intermediates [30,40,113]. So-called deletor mice, expressing a disease-causing mutant variant of TWINKLE, have also been shown to develop OXPHOS deficiencies- symptoms typically associated with PEO [114]. Since TWINKLE activity is dependent on the continuous cycling through individual subunits within the hexamer, the presence of a single inactive mutant subunit would be sufficient to stall the catalytic cycle. This may explain why many of these PEO-associated mutations exhibit a dominant effect in patients.

Despite significant advances in our understanding of the molecular basis of PEO, it is still difficult to systematically correlate (1) the location of the mutated residue or (2) the severity of the enzymatic defects in our in vitro assays with the symptoms presented in affected patients. This presents a challenge when attempting to predict the severity of de novo PEO-associated mutations and necessitates a case-by-case analysis.

#### 2.2.2. Other Diseases

Mutations in the TWINKLE helicase are also responsible for a wide range of disorders in addition to PEO. Perrault disease, mtDNA depletion syndromes (MDSs) and infantile-onset spinocerebellar ataxia (IOSCA) are all caused by recessively-inherited mutations in TWINKLE.

mtDNA depletion syndromes are a phenotypically heterogeneous set of autosomal recessive disorders which include both hepatocerebral mtDNA depletion syndrome and IOSCA [115,116,117]. Several mutations in the *TWNK* gene are associated with MDSs/IOSCA, most notably: A318T, T457I and Y508C (Figure 4). These mutations lead to severe mtDNA depletion and impaired OXPHOS activity and manifest clinically as seizures, developmental delay and peripheral neuropathy [98,100]. Although little in vitro biochemistry using these mutants has been performed, structural modelling suggests that T457 lies in the NTP-binding pocket of TWINKLE, while Y508 forms a pocket for I367 in the linker region on the neighboring subunit [118]. Disruption to NTP binding/hydrolysis and/or oligomerization would result in severe impairment of TWINKLE activity and correlates well with the observed mtDNA depletion phenotypes in patients.

Perrault syndrome is another disease associated with recessive mutations in TWINKLE. It is characterized by sensorineural hearing loss, abnormal ovaries, ataxia, muscle weakness and intellectual disability, although these symptoms vary among patients [119,120]. Several mutations have been linked to the disease, although R391H, W441G, V507I and N585S appear to be the most widely-reported (Figure 4) [121,122]. Structurally, V507 likely interacts with I367 in the linker helix of an adjacent subunit (similar to that seen for Y508 in IOSCA). Perhaps most interestingly, however, is N585, which lies in close proximity to both the conserved arginine finger R609 in the NTP-binding site as well as to bound ssDNA (Figure 5). The N585S mutation, which substitutes a bulky asparagine residue for a small serine, would very likely result in both impaired ATPase and ssDNA-binding activity. In addition, structural modelling suggests that R391 is also located within the NTP-binding site, while W441 is located in close proximity to the bound ssDNA (Figure 5).

TWINKLE mutations have also been linked to a number of ataxia neuropathy spectrum disorders, including mitochondrial recessive ataxia syndrome (MIRAS) and sensory ataxia neuropathy dysarthria and ophthalmoplegia (SANDO) [98,99]. These phenotypes are typically associated with mutations in POLγ rather than in TWINKLE, highlighting the clinical overlap seen in many mitochondrial disorders. Finally, there are also a large number of reported rare variants in the *TWNK* gene for which the significance of pathogenicity is uncertain (Figure 4). While many of these are likely benign, they do present an opportunity for further biochemical characterization. As more disease-causing TWINKLE variants are discovered, the need for consolidated structure–function studies becomes greater. We believe such studies will not only further our understanding of the molecular basis of TWINKLE-associated mitochondrial diseases but will also be key to identifying therapeutic targets and treatment strategies in the future.

## 3. Other Potential Mitochondrial DNA Helicases

**Figure 6:**
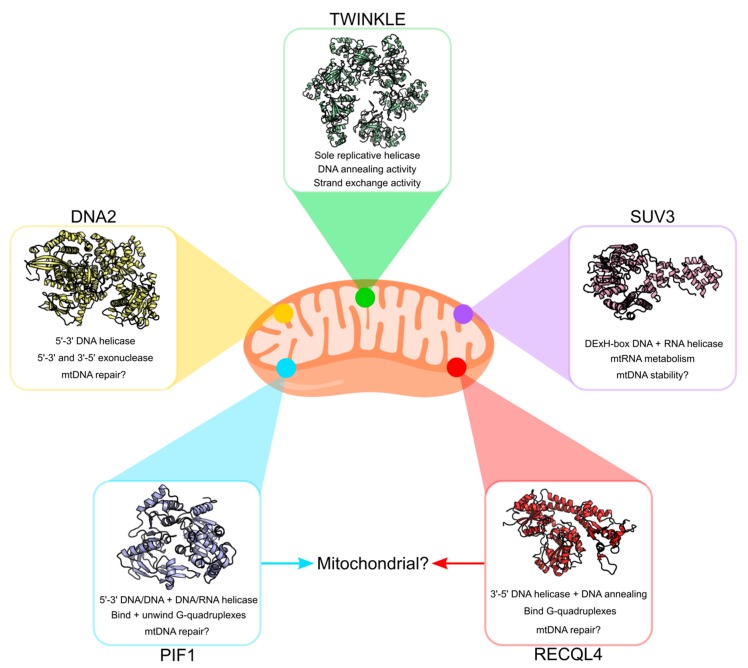
Potential DNA helicases in human mitochondria. In addition to the TWINKLE helicase, human mitochondria possess a number of additional helicases with unknown/poorly understood function. These include PIF1 (PDB ID: 6HPH), DNA2 (PDB ID: 5EAW), RECQL4 (PDB ID: 5LST) and SUV3 (PDB ID: 3RC3). Although some evidence exists for the presence of PIF1 and RECQL4 in human mitochondria, the mitochondrial localization and role of PIF1 and RECQL4 remains debated.

Although TWINKLE remains the most studied mitochondrial helicase in humans, a number of additional helicases have been proposed to localize to mammalian mitochondria (Figure 6). These include *h*PIF1 [123], *h*DNA2 [54], *h*RECQL4 [124] and *h*SUV3 [125]. The majority of these are homologues of yeast mitochondrial helicases, although preliminary studies suggest functional differences from their yeast counterparts. Whether or not all of these helicases actually exist in human mitochondria and what roles they might play is still a topic of debate. We conclude this review with a brief overview of these helicases and their putative roles in mtDNA maintenance. For a more comprehensive overview of these helicases, we recommend the reviews by de Souza-Pinto et al. ([126]) and Szczesny et al. ([127]).

### 3.1. PIF1 Helicase

The PIF1 protein belongs to the SF1 helicase superfamily and displays 5ʹ–3ʹ helicase activity on both DNA/DNA and DNA/RNA hybrids in vitro, albeit poorly processive [123,128,129]. A recent review by Byrd and Raney ([130]) gives a detailed account of the structure and putative functions of PIF1. In yeast, the PIF1 gene encodes a nuclear and mitochondrial isoform (*y*PIF1) starting at alternative translational start sites with a mitochondrial target signal (MTS) site between them [131]. Similarly, alternative start sites for the PIF1 gene have been suggested to exist in humans (*h*PIF1) [132]. In addition, an alternative splice variant, called *h*PIF1β, with a putative MTS at its C-terminus has also been proposed to exist in human cells [121]. The exact function of *h*PIF1 in human mitochondria is still unclear. Although *y*PIF1 is crucial for mtDNA maintenance in fission yeast, *h*PIF1 is not essential for mtDNA maintenance [133]. Nevertheless, *h*PIF1 has been demonstrated to preferentially bind and unwind G-quadruplex structures in vitro [134,135,136,137]. If active in human mitochondria, it probably plays a role in resolving such structures preventing replication stalling [137]. That *h*PIF1 plays a role in human mitochondria is also supported by in vivo studies where PIF1 knockout mice develop a mitochondrial myopathy with respiratory chain deficiencies [138].

### 3.2. DNA2 Nuclease/Helicase

Yeast DNA2 is a bifunctional protein comprising a RecB nuclease domain and a C-terminal helicase domain. It shows 5ʹ–3ʹ DNA-dependent helicase activity as well as ATPase and endonuclease activities [139,140,141,142]. The human variant is less well-characterised but is thought to possess these functions in mitochondria [143]. *h*DNA2 localizes to mitochondria and has been shown to colocalize to mtDNA and TWINKLE, suggesting a role in mtDNA maintenance [54,144,145]. It also stimulates POLγ activity in vitro as well as supporting primer removal and ligation of mtDNA in mitochondrial extracts [54,146]. Mutations in *h*DNA2 have been implicated in an adult-onset mitochondrial myopathy characterized by mtDNA instability [147]. A homozygous *h*DNA2 knockout has also been shown to be embryonically lethal in mice, although this was not explicitly linked to mitochondrial dysfunction per se [146]. In addition, *h*DNA2 has been proposed to be involved in long-patch base excision repair (LP-BER) [53]. It is even an attractive candidate for cancer therapy, given reports that it is overexpressed in various cancers [148]. Taken together, *h*DNA2 likely plays several roles in mitochondria including in mtDNA repair.

### 3.3. RECQL4 Helicase

The RecQ helicases are a group of ATP-dependent SF2 helicases that catalyze the 3ʹ–5ʹ unwinding of DNA/DNA duplexes and play roles in DNA repair and replication [124,149]. Thus far, only one member of the RecQ family (RECQL4) has been suggested to contain an MTS and localize to mitochondria [150,151]. However, definitive evidence for its presence and activity in mitochondria is still lacking. RECQL4 is related to the yeast replication factor Sld2 and is thought to be able to bind Holliday junctions and G-quadruplex DNA as well as possess DNA annealing activity [152,153,154,155]. RECQL4 levels slightly affect mtDNA copy number and a weak interaction between RECQL4 and TWINKLE has been reported in human whole-cell extracts [156,157]. However, although mutations in RECQL4 are associated with a number of disorders, none have yet been linked to defects in mtDNA replication/maintenance [158,159]. This raises questions about what role this protein plays in mitochondria.

### 3.4. SUV3 and Other RNA Helicases

Human SUV3 is an ATP-dependent DExH-box DNA and RNA helicase which shares a high degree of sequence similarity with its yeast homolog *y*Suv3 [125]. It has been shown to localise to mitochondria where it associates with the mitochondrial nucleoid [160,161,162]. Together with the polynucleotide phosphorylase (PNPase), *h*SUV3 forms a degradasome which is responsible for the degradation of mitochondrial RNAs [163,164]. Knockout of *h*SUV3 is embryonically lethal and is associated with reduced mtDNA copy number and an elevated mtDNA mutation load [165,166]. It is still unclear how defects in RNA degradation contribute to mtDNA instability. However, it has been demonstrated that *h*SUV3 preferentially unwinds dsDNA rather than dsRNA suggesting that *h*SUV3 might have an additional function in mtDNA maintenance. In addition to SUV3, a number of other RNA helicases are also present in mitochondria and include the DExD-box helicase DDX28 and DExH-box helicase DHX30 [167,168]. The functions of these helicases are still not fully understood, although they appear to be involved in mitoribosome assembly [169,170]. Whether these helicases are also involved in mtDNA maintenance is, as yet, unknown.

## 4. Future Perspectives and Conclusions

Nearly 20 years have passed since the discovery of the TWINKLE helicase. Despite significant advances in our understanding of mtDNA replication, there remain many gaps in our knowledge regarding the role(s) played by TWINKLE and other mitochondrial helicases. Potential new partners of the mtDNA replication/maintenance machinery are constantly being identified by techniques such as BioID, making this an exciting time for the field of mitochondrial biology. The advent of cryo-EM and the potential to solve the structure of the human mitochondrial replisome is also of particular interest to our group. We believe that this will provide a multitude of answers to questions which we have, as yet, been unable to provide. These include the architecture of the replisome, the structural basis for disease-causing mutations and an insight into the mechanistic coupling of TWINKLE helicase activity to mtDNA synthesis and maintenance.
